# Three-dimensional Contrast-enhanced Ultrasound in Response Assessment for Breast Cancer: A Comparison with Dynamic Contrast-enhanced Magnetic Resonance Imaging and Pathology

**DOI:** 10.1038/srep33832

**Published:** 2016-09-22

**Authors:** Wan-Ru Jia, Lei Tang, Deng-Bin Wang, Wei-Min Chai, Xiao-Chun Fei, Jian-Rong He, Man Chen, Wen-Ping Wang

**Affiliations:** 1 Rui Jin Hospital, Shanghai Jiao Tong University School of Medicine, Department of Diagnostic Ultrasound, Shanghai, 200025, China; 2 Xin Hua Hospital, Shanghai Jiao Tong University School of Medicine, Department of Radiology, Shanghai, 200092, China; 3 Rui Jin Hospital, Shanghai Jiao Tong University School of Medicine, Department of Radiology, Shanghai, 200025, China; 4 Rui Jin Hospital, Shanghai Jiao Tong University School of Medicine, Department of Pathology, Shanghai, 200025, China; 5 Rui Jin Hospital, Shanghai Jiao Tong University School of Medicine, Department of Comprehensive breast health center, Shanghai, 200025, China; 6 Zhong Shan Hospital, Fu Dan University School of Medicine, Shanghai, 200025, China

## Abstract

To compare the capabilities of three-dimensional contrast enhanced ultrasound (3D-CEUS) and dynamic contrast-enhanced magnetic resonance (DCE-MRI) in predicting the response to neoadjuvant chemotherapy (NAC) among breast cancer patients, 48 patients with unilateral breast cancer were recruited for 3D-CEUS and DCE-MRI examinations both before and after NAC; pathology was used to validate the results. This study was approved by the institutional review board, and written informed consent was obtained from each patient. Imaging feature changes and pathological vascularity response, including microvessel density (MVD) and vascular endothelial growth factor (VEGF), were calculated. Pathological complete response (pCR) and major histological response (MHR) were used as references. The 3D-CEUS score, DCE-MRI score, MVD and VEGF significantly decreased (*P* < 0.0001) after NAC. The correlations between Δ3D-CEUS and ΔDCE-MRI with pCR (r = 0.649, *P* < 0.0001; r = 0.639, *P* < 0.0001) and MHR (r = 0.863, *P* < 0.0001; r = 0.836, *P* < 0.0001) were significant. All scores showed significant differences between the pCR and non-pCR groups with folder changes of 0.1, 0.1, 2.4, and 2.3, respectively (*P* = 0.0001, <0.0001, <0.0001 and <0.0001). In conclusion, 3D-CEUS is effective in assessing the response of breast cancer patients undergoing NAC.

Breast cancer is the most common cancer affecting females worldwide. The American Cancer Society announced that approximately 232,340 new cases of invasive breast cancer and 39,620 breast cancer deaths were expected to occur among women in the United States in 2013^1^. For locally advanced breast cancer patients, neoadjuvant chemotherapy (NAC) has been increasingly applied in personalized therapy^2^,^3^, and a good response to NAC with a complete pathologic response (pCR) is a surrogate marker for better overall survival and disease-free survival^2^,^4^, whereas a major histological response (MHR) indicates a high sensitivity to chemotherapy drugs^5^. However, pathological assessments have a time lag and a traumatic defect. Because the metabolic response has been reported to precede morphologic changes during therapy^6^, the clinical value of image evaluation on the basis of traditional RECIST criteria is also limited^7^,^8^,^9^,^10^. Because it clearly characterizes both the tumour morphology and vascularity, dynamic contrast-enhanced magnetic resonance imaging (DCE-MRI) has been widely used for response assessment, but over- or underestimation is still inevitable^11^,^12^,^13^.

Folkman *et al*. emphasized the importance of angiogenesis in tumour growth and proposed that the inhibition of angiogenesis might arrest solid tumours, so the accurate evaluation of vascular characteristics during therapy remains an important topic^14^,^15^. Vascular endothelial growth factors (VEGFs) are important cytokines in regulating endothelial cell mitosis, proliferation and functional development, and microvessel density (MVD) has been the gold standard for the evaluation of tumour angiogenesis^15^,^16^,^17^,^18^. Targeted at tumour angiogenesis and vascularity, contrast enhanced ultrasound (CEUS) has been recommended to assess tumour response, including that of gastrointestinal stromal tumours and renal cell carcinoma^19^,^20^,^21^, and molecular ultrasound imaging can be used to detect early tumour responses to antiangiogenic therapy using targeted contrast agents^22^. Three-dimensional contrast enhanced ultrasound (3D-CEUS), which can objectively depict tumour vascularity and intratumoural perfusion by reconstructing stereoscopic images^23^,^24^, has been reported to produce satisfactory results for the evaluation of treatment response in liver cancer patients after local therapies^25^. In former studies, we found that 3D-CEUS characteristics were significantly different between benign and malignant breast tumours^24^, including the presence of penetrating vessels, rim perfusion, degree of dilation and the peripheral vessel courses. Therefore, a more objective 3D-CEUS score system, which comprehensively analysed the former features, was created for evaluating breast tumour angiogenesis^23^, and if 4 points were used as the cut-off value, the diagnostic accuracy was similar to DCE-MRI in terms of breast tumour differentiation. However, to the best of our knowledge, 3D-CEUS has never been reported to predict the pathological response of breast cancer patients treated with NAC, utilizing pathology as evidence of the test results. Therefore, the purpose of this study is to explore the effectiveness of using semi-quantified 3D-CEUS enhancement patterns for the response assessment of breast cancer patients in a comparison with DCE-MRI.

## Results

### Patient characteristics and pathological responses to NAC

A total of 48 patients with a median age of 47.08 years (range, 28–63 years) with unilateral breast cancer were recruited in our study; among which, 32 women were premenopausal. All patients received full cycles of NAC followed by surgery. Forty-seven patients had invasive ductal carcinoma, and the other patient had invasive micropapillary carcinoma. Using the Miller and Payne grading system, 28 patients were categorized as grade 3 after NAC, 12 were grade 4, and 8 were grade 5. Therefore, 8 patients with grade 5 achieved pCR, whereas 20 patients achieved MHR with grade 4 or 5. Except for the 8 patients who achieved pCR, all biopsy tissues and surgical specimens of the remaining 40 patients were processed for MVD and VEGF staining.

### Imaging and pathology evaluation before and after NAC treatment

The changes in the 3D-CEUS score and DCE-MRI score before and after NAC treatment are presented in Table 1. Among the 48 patients, the mean 3D-CEUS score was 7.5 ± 1.6 and 3.3 ± 2.1 before and after NAC, respectively, and the difference was 4.2 ± 2.3 (P < 0.0001), whereas the mean DCE-MRI score decreased from 7.5 ± 1.2 to 3.3 ± 2.2 with a significant difference (P < 0.0001). For all 40 patients with a pathology evaluation, the MVD count was (12 ± 3.3) * 10^3^ and (7.8 ± 2.2) * 10^3^ before and after NAC, respectively, which were significantly different (P < 0.0001), and the VEGF score decreased from 5.6 ± 0.6 to 3.8 ± 0.9, which was also significantly different (P < 0.0001). The fold changes were 0.4, 0.4, 0.6, and 0.7 for each of them (Table 1).

### Correlation between the imaging and pathological vascularity characteristics

Table 2 shows the correlation between the image scores and microvessel quantification among 40 breast patients with a pathology evaluation. Before NAC, the 3D-CEUS score was significantly correlated with MVD (r = 0.729, P < 0.0001) and VEGF (r = 0.454, P = 0.0046), but the DCE-MRI score was not (MVD r = −0.125, P = 0.4340 & VEGF r = −0.247, P = 0.1235). After NAC, the 3D-CEUS score was statistically associated with MVD (r = 0.549, P = 0.0006) and VEGF (r = 0.460, P = 0.0041), but the DCE-MRI score was not (MVD r = -0.110, P = 0.4925 and VEGF r = −0.054, P = 0.7365). However, for the difference before and after NAC, only the Δ3D-CEUS was correlated with VEGF (r = 0.408, P = 0.0108) (Table 2).

### Correlation between the image score and treatment response

Of the 48 patients, the correlation between treatment response (pCR and MHR) and the image score is shown in Table 3. After NAC, both the 3D-CEUS and DCE-MRI scores were correlated with pCR (r = −0.573, P = 0.0001; r = −0.615, P < 0.0001) and MHR (r = −0.541, P = 0.0002; r = −0.702, P < 0.0001), and the Δ3D-CEUS and ΔDCE-MRI scores were correlated with pCR (r = 0.649, P < 0.0001; r = 0.639, P < 0.0001) and MHR (r = 0.863, P < 0.0001; r = 0.836, P < 0.0001) (Table 3).

### Performance of the imaging changes and pathological vascularity changes in predicting pCR and MHR

In a comparison of the pCR group and non-pCR group, all 3D-CEUS scores after NAC, the DCE-MRI score after NAC, and the Δ3D-CEUS and ΔDCE-MRI were significantly different (P = 0.0001, <0.0001, <0.0001, and <0.0001, respectively), and the folder changes were 0.1, 0.1, 2.4, and 2.3, respectively. The associated AUC ranged from 0.934 to 0.991. When the cut-off points of the 3D-CEUS score after NAC were <1 and the Δ3D-CEUS score were >6, respectively, the sensitivity, specificity, PPV, and NPV were 88%, 100%, 100% and 98%, respectively; when DCE-MRI scores were <1, they were 88%, 98%, 88% and 98%. respectively; and when ΔDCE-MRI scores were >5, they were 100%, 95%, 80% and 100%, respectively. Among the 20 patients who achieved MHR and the 28 patients with NMHR (non-major histological response), the sensitivity, specificity, PPV, and NPV were significantly different (P = 0.0003, <0.0001, <0.0001, and <0.0001), with folder changes of 0.5, 0.3, 2.5, and 2.4, respectively. The associated AUC ranged from 0.810 to 0.993. Among the 12 patients with MHR and 28 patients with NMHR, the ΔMVD and ΔVEGF did not significantly differ (P = 0.2497 and P = 0.0766, respectively) (Table 4).

## Discussion

Angiogenesis is crucial for autonomous tumour growth, invasion and metastasis^14^. The overgrowth of tumour neovascularization can cause high vascular permeability owing to weak vascular walls with only a layer of endothelial cells and thus leads to significant enhancement. The mechanisms of antiangiogenesis in NAC are various; the agents we have used in this study worked in different stages of the cell cycle, leading to cell degeneration and apoptosis, followed by tumour shrinkage; consequently, intratumoural enhancement was more apparent than at the periphery. Colour Doppler has been used to prove that the presence of arterial signals indicates a persistent viable tumour, whereas undetectable colour signals indicate successful tumour treatment^26^,^27^. Kedar *et al*. even found that colour Doppler flow changes occurred at least 4 weeks before changes in clinical and B-mode US examination in 40% and 38% of cases, respectively^26^. Palmowski *et al*. then indicated that immature intratumoural vessels degraded markedly upon therapy, whereas large mature vessels on the tumour periphery were more therapy resistant and drew closer owing to tumour shrinkage, and contrast-enhanced ultrasound (CEUS) can provide evidence of vascular maturity in tumours^27^. Therefore, CEUS has already been proven as an effective indicator of breast cancer response^28^,^29^.

The combined advantages of CEUS and three-dimensional ultrasound, 3D-CEUS, can evaluate tumour vascularity in a three dimensional field and can thus be utilized for breast tumour differentiation. Luo *et al*. has found that 3D-CEUS was useful in the evaluation and characterization of vascular patterns of focal liver tumours^30^. Xu *et al*. has described the advantages of 3D-CEUS in the evaluation of 107 lesions in 95 consecutive patients with liver cancer who underwent local therapies, and found that 3D-CEUS not only enhanced the diagnostic confidence in the majority of the patients but also changed the management of some patients^25^. In previous studies, we identified some characteristics of peripheral and intratumoural vessels in malignant breast tumours^24^, and scored these features 0–2 points^23^. Encouragingly, the 3D-CEUS score system displayed inspiring diagnostic performance and good agreement with DCE-MRI^23^. DCE-MRI is a highly sensitive imaging modality for early therapy assessment, residual lesion characterization and recurrence prediction for breast cancer with post-enhancement evaluation advantages^11^,^12^,^13^. In this study, both the 3D-CEUS and DCE-MRI scores were significantly correlated with pCR and MHR, respectively. Δ3D-CEUS was most strongly correlated with MHR, followed by ΔDCE-MRI. Between the pCR group and the non-pCR group, the specificity and PPV of 3D-CEUS and Δ3D-CEUS were 100%, whereas the sensitivity and NPV of ΔDCE-MRI were 100%, suggesting the potential application of 3D-CEUS. Similarly, between the MHR group and the NMHR group, the predictive performances of 3D-CEUS and DCE-MRI were better than those of ΔMVD and ΔVEGF. Δ3D-CEUS obtained a very high sensitivity, specificity and AUC, from which we can conclude that 3D-CEUS was comparable to MHR in the evaluation of the response to NAC among breast cancer patients.

All 40 patients who did not achieve pCR underwent MVD and VEGF staining, and we found that both MVD and VEGF decreased after the completion of NAC. Sun *et al*. hypothesized that, for patients with small HCC, the MVD level was an independent predictor of disease-free survival^17^. Hanahan *et al*. identified angiogenic switch mechanisms during tumorigenesis and thought that angiogenesis was regulated by both vascular promoting factors and inhibition factors^18^. VEGF have been found to be important cytokines in regulating endothelial cell proliferation and function development, which may also influence the expression of MVD^16^. Therefore, angiogenesis can be indirectly evaluated by the MVD and VEGF staining of biopsy tissue or resected specimens. When we made use of 3D-CEUS scoring to depict the vascular and intratumoural perfusion characteristics in a comparison with the DCE-MRI score, the results of this study were promising, with significant Δ3D-CEUS and ΔDCE-MRI scores for the prediction of pCR and MHR, and these changes were also correlated with MVD and VEGF changes to an extent. In our studies, we found that 3D-CEUS was more related to VEGF and MVD than DCE-MRI before treatment, which suggested the potentials of 3D-CEUS in the prediction of NAC response of breast cancer patients. However, after NAC, the correlation between 3D-CEUS and MVD declined, whereas DCE-MRI was similarly correlated to an extent. The tumour contractures and cell flinch after chemotherapy, which affect the display of vascular characteristics within the tumour, and the surrounding tumour fibrosis and necrosis may explain this result. Further investigations of large samples are necessary for the clinical application of this method in the future.

There were some limitations in our studies. First, difficulties existed in selecting patients who were willing to accept both 3D-CEUS and DCE-MRI examination, for they were not only expensive but also time-consuming, so the samples were relatively small, and the conclusions may be overestimated. Further investigations of large samples with various histological and molecular subtypes are needed. Second, the 3D-CEUS images were less panoramic than DCE-MRI, so the 3D-CEUS index was subjectively observed, and interobserver and intraobserver variability in image interpretation has not been evaluated. Third, breast MR imaging was performed using a dedicated breast magnetic resonance imaging (DBMRI) system in our institution, so the reproducibility for whole body instruments is worth further discussion. Finally, the the microvessels were quantified by the counting procedure of Weidner *et al*.^31^,^32^, so the results may be influenced by selection of ‘hot spots’.

In conclusion, we demonstrated that 3D-CEUS had the potential to predict NAC treatment response in patients with breast cancer compared to DCE-MRI and pathology, which may provide a reliable foundation for comprehensive assessments and personalized treatment strategies for breast cancer patients.

## Methods

### Patients

This study was approved by the institutional review board of Ruijin Hospital, Shanghai Jiaotong University School of Medicine, and informed consent was obtained from all patients. All methods were carried out in accordance with the relevant guidelines. Between March 2010 and February 2012, patients from the same institution who had primary breast cancer histologically diagnosed by core needle biopsy were recruited to undergo 3D-CEUS and DCE-MRI examinations before and after NAC. The eligibility criteria were as follows: (a) age of 18–70 years, without any history of treatment for breast cancer before NAC, (b) had no contraindications to chemotherapy, CEUS and DCE-MRI, and (c) patients who underwent 3D-CEUS and DCE-MRI examinations before and after NAC. During this period, 59 patients were recommended for NAC, and 11 patients were excluded for the following reasons: 6 patients were lost to follow up, 2 patients had contraindications for CEUS or MRI, and 3 patients had inadequate or unsatisfactory imaging information. A total of 48 female patients (mean age, 47.08 years; age range, 28 ~ 63 years) were included in this study. All patients were clinically staged II or III^33^. Twenty patients received 5-fluorouracil (500 mg/m^2^), epirubicin (100 mg/m^2^) and cyclophosphamide (60 mg/m^2^) every three weeks for four cycles, and 28 patients received paclitaxel (175 mg/m^2^), epirubicin (60 mg/m^2^) and cyclophosphamide (60 mg/m^2^) every three weeks for six cycles. Ten HER-2 positive patients received weekly herceptin (initial dose, 4 mg per kilogram of body weight; subsequent dose, 2 mg/kg) for 6 months.

### US and 3D-CEUS Examination

Conventional US, CEUS, and 3D-CEUS scanning were performed using the same ultrasound machine Mylab 90 (Esaote, Genoa, Italy). Conventional US and colour Doppler US were performed using a LA 523 transducer with a frequency of 13–4 MHz, whereas 2D-CEUS was evaluated using a LA 522 transducer with a frequency of 9–3 MHz. The BL 433 volume transducer with a frequency of 15–9 MHz was used for 3D scanning and 3D-CEUS. The contrast agent was SonoVue (BR1, Bracco SpA, Milan, Italy), a sulfur hexafluoride-filled microbubble contrast agent.

First, conventional US, colour Doppler US and baseline 3D scanning were performed by one radiologist (M. C., 5 years of experience in breast CEUS and one year of experience in breast 3D-CEUS) to observe the general features of the breast tumours and to select the best tumour imaging in the maximum plane, from which both the tumours and the normal adjacent breast tissue could be observed. Additionally, the appropriate volume angle was defined so that the whole lesion would be included in the volume data without signal loss. The imaging parameter settings were optimized for high quality images, and the longest diameters were measured. According to the breast imaging reporting and data system (BI-RADS-US)^34^,^35^, for lesions without circumscribed margins, the angular, microbulated, and spiculated margins would also be included in the measurement, whereas for multifocal lesions, the largest one was selected for measurement. Subsequently, CEUS was first performed using a LA 522 transducer with a frequency of 9–3 MHz. Then, we performed a flush to destroy remaining microbubbles, and 3D-CEUS was initiated when the signals from the microbubbles in the large vessels, such as the axillary vein, disappeared. The same dose of the contrast agent SonoVue used in CEUS was injected (2.4 mL of SonoVue as a bolus through an antecubital vein, followed by a flush of 5 mL of 0.9% saline). Ten seconds later, 3D-CEUS images were continuously obtained more than five times for a total time over 2 minutes. The imaging settings for 3D-CEUS were as follows: MI, 0.08–0.13; one focal zone; power output, 3–6%; dynamic range, 40–60 dB; and volume angle, 30–50°. The transducer was kept in a stable position without movement during the scanning, and the patient was asked to hold their breath. All the data, including the CEUS and 3D-CEUS images, were stored on the hard disk of the ultrasonography machine in the DICOM format for further analysis.

### DCE-MRI Examination

A dedicated breast magnetic resonance imaging (DBMRI) system (Aurora Dedicated Breast MRI Systems, USA) with a dedicated breast coil was used for breast MR imaging. The contrast agent we used was Gd-DTPA (Magnevist, Germany), which was intravenously injected as a bolus (1.5 mL/s) at a dose of 0.2 mmol/kg body weight followed by a 20 mL saline solution flush. A dynamic series of axial T1-weighted fat-suppression images (TR 29 ms, TE 4.8 ms, slice thickness 1.1 mm, matrix: 360 × 360 × 128, FOV 36 cm), including one pre- and four post-contrast scans, were obtained after a localizer on the axial image and coil calibration. The dynamic images were acquired 90 s after the contrast media injection. The scan time was three minutes per scan, and the total time was 12 min.

### Quantification of the enhancement patterns on 3D-CEUS and DCE-MRI

**Figure 1:**
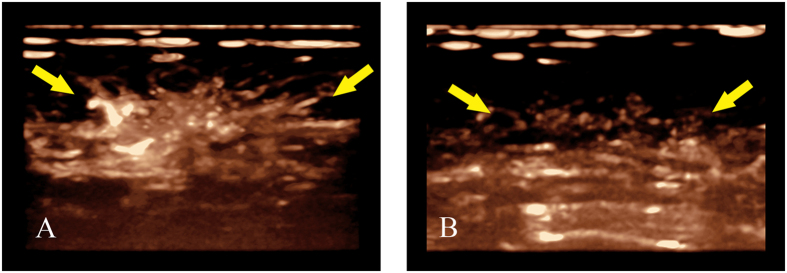
(**A**,**B**) Changes of peripheral vessels displayed by 3D-CEUS before and after completion of NAC for breast cancer (female, 64 years old, invasive ductal carcinoma, grade II); (**A**) Before NAC, 3D-CEUS image showed coarse peripheral vessels with radial distribution (arrow) at the strongest stage of perfusion. (**B**) After completion of NAC, radial peripheral vessels (arrow) disappeared at the strongest stage of perfusion.

**Figure 2:**
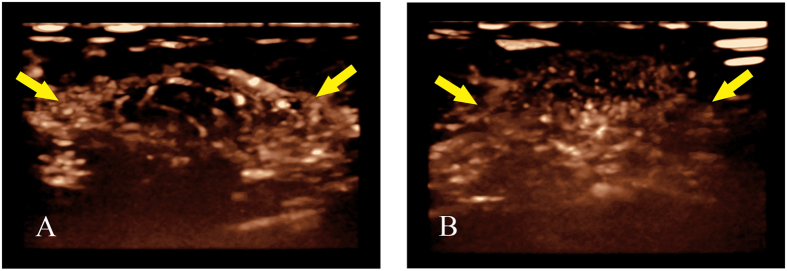
(**A**,**B**) Changes of rim perfusion displayed by 3D-CEUS before and after completion of NAC for breast cancer (female, 45 years old, invasive ductal carcinoma, grade II); (**A**) Before NAC, 3D-CEUS image showed coarse rim perfusion (arrow) at the strongest stage of perfusion. (**B**) After completion of NAC, rim perfusion (arrow) disappeared at the strongest stage of perfusion.

Two investigators who did not perform US examinations and who were blind to the surgical and histological information retrospectively analysed these images (L.T. and W.R.J.; one year of experience each in breast 3D-CEUS). Conclusions were obtained after independent interpretations, and a consensus was achieved if disagreement existed. Based on our clinical experience and previous studies^23^,^24^, a score from 0 to 2 was given for each characteristic: (a) peripheral vessels and their distribution (radial or not), (b) courses and the degree of dilation of peripheral vessels (distorted or not, coarse or not), (c) penetrating vessels and their courses (running inside the tumour or towards the centre), (d) rim perfusion and degree (thin/moderate or coarse), and (e) intratumoural vessels and their degree of dilatation (coarse or not) (Table 5, Figs 1 and 2). All scores were recorded before and after the completion of NAC for further comparisons.

**Figure 3:**
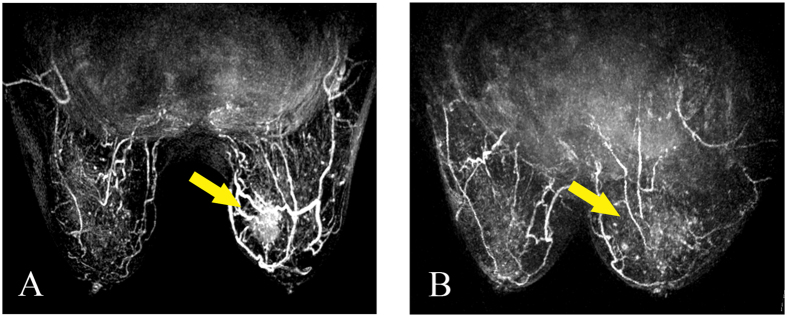
(**A**,**B**) Peripheral vessel changes on DCE-MRI before and after completion of NAC for breast cancer (female, 64 years old, invasive ductal carcinoma, grade II). (**A**) Before NAC, DCE-MRI image showed apparently richer and coarser peripheral vessels of the tumor. (**B**) After the completion of NAC, the peripheral vessels and the tumor seemed to disappear compared to the contralateral breast.

**Figure 4:**
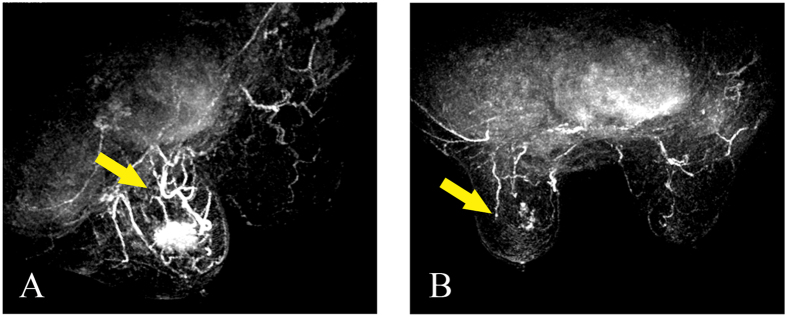
(**A**,**B)** Penetrating vessel change on DCE-MRI before and after completion of NAC for breast cancer (female, 57 years old, invasive ductal carcinoma, grade III). (**A**) Before NAC, DCE-MRI image showed coarse penetrating vessels compared to the contralateral breast. (**B**) After the completion of NAC, the penetrating vessels and the tumor disappeared.

The MRI images were also interpreted by two off-sight breast radiologists without any information of the patient cases (D.B.W. and W.M.C, each with more than 5 years of experience). Image post-processing was performed using a dedicated AURORA CAD workstation. The tumour area with the highest signal intensity enhancement within the tumour was selected as the region of interest (ROI). Each reader individually analysed the MRI characteristics and the enhancement pattern, and a consensus was achieved if necessary. Additionally, a score of 0 to 2 points was also given for the enhancement feature according to our former publications: (a) enhancement pattern (homogeneous, heterogeneous and rim enhancement); (b) TIC curve (type I/persistent, type II /plateau, type III/wash-out); (c) initial signal increase (<50%/mild enhancement, 50–100%/moderate enhancement, >100%/significant enhancement); (d) peripheral vessels and their degree of dilation (not rich, richer, richer and coarser); and (e) penetrating vessels (present or not) and their degree of dilation (thin/moderate and coarse/encircling)^23^,^24^ (Figs 3 and 4). The differences between the 3D-CEUS and DCE-MRI scores before and after the completion of NAC were recorded as the Δ3D-CEUS and ΔDCE-MRI scores.

### Pathological Treatment Response Evaluation

**Figure 5:**
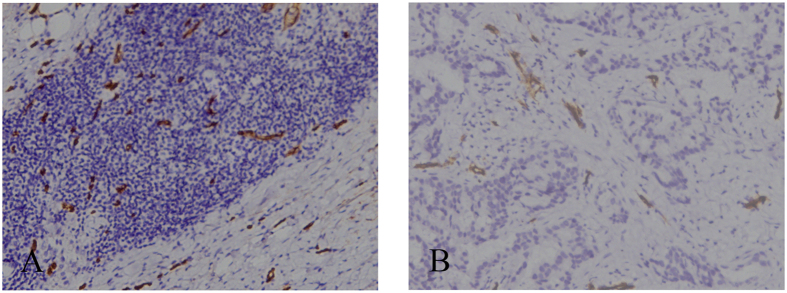
(**A,B**) MVD changes before and after the completion of NAC for breast cancer (female, 31 years old, invasive ductal carcinoma, grade II). (**A**) Before NAC, core-needle biopsy tissue showed high MVD distribution. (**B**) After the completion of NAC, MVD distributed lower in surgical specimens of the same patient (CD34 staining, original magnification×100).

**Figure 6:**
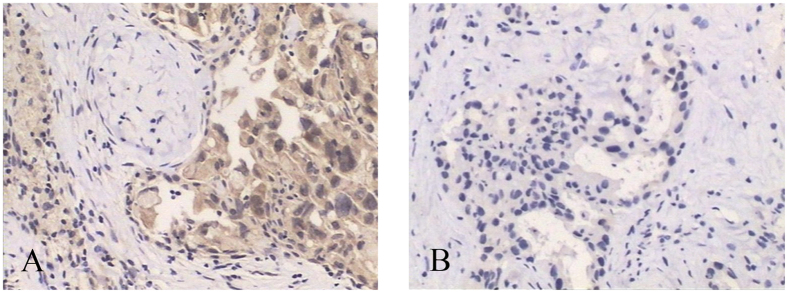
(**A**,**B**) VEGF expression changes before and after the completion of NAC for breast cancers (female, 57 years old, invasive ductal carcinoma, grade III). (**A**) Before NAC, core-needle biopsy tissue showed high VEGF expression. (**B**) After the completion of NAC, low VEGF expression from surgical specimens of the same patient (IHC, original magnification×100).

Pathological diagnosis and response to NAC were analysed by an experienced breast pathologist (X.C.F., 10 years of experience in breast pathology) according to the Miller and Payne system, which included 5 grades based on malignant cell changes between the surgical specimen and biopsy tissue^36^. Grade 1: No change or some alteration to individual malignant cells but no reduction in overall cellularity; Grade 2: A minor loss of tumour cells but overall cellularity still high, up to 30% loss; Grade 3: 30–90% loss of malignant cells; Grade 4: More than 90% loss of malignant cells; and Grade 5: No malignant cells identifiable in sections from the site of the tumour with or without ductal carcinoma *in situ*. Of note, pathological complete response (pCR) was defined as grade 5. We further classified major histological response (MHR), including grade 4 and 5, and non-major histological response (NMHR)^5^. On the other hand, we quantified MVD according to the counting procedure described by Weidner *et al*.^31^,^32^. VEGF expression were determined by immunohistochemistry using a rabbit polyclonal anti-VEGF antibody (BioGenex, USA)^37^,^38^. The ΔMVD and ΔVEGF scores were also recorded by comparing their changes both before and after NAC (Figs 5 and 6).

### Statistical Analysis

Statistical analyses were performed using SPSS version 17.0 software for Windows (SPSS Inc., Chicago, IL). χ^2^ tests were used to compare variables, and a value of *P* < 0.05 was considered to be statistically significant. Histological pCR and MHR were used as reference standards for the calculations. The folder change was calculated for the rate of change. The correlation between the imaging and histological analysis, the Δ3D-CEUS, ΔDCE-MRI score and ΔMVD, and the ΔVEGF score were calculated using Spearman coefficients *(r* values), which were classified as low (|*r*|: 0.3–0.5), significant (|*r*|: 0.5–0.8), and high correlations (|*r*|: 0.8–1.0). The receiver operating characteristic curve was used to determine the sensitivity, specificity, positive predictive value (PPV) and negative predictive value (NPV) of 3D-CEUS and DCE-MRI scores in predicting pCR and MHR by comparing the area under the receiver operating characteristic curves (AUC).

## Additional Information

**How to cite this article**: Jia, W.-R. *et al*. Three-dimensional Contrast-enhanced Ultrasound in Response Assessment for Breast Cancer: A Comparison with Dynamic Contrast-enhanced Magnetic Resonance Imaging and Pathology. *Sci. Rep.* **6**, 33832; doi: 10.1038/srep33832 (2016).

## Figures and Tables

**Table 1:** Imaging and pathology evaluation before and after NAC treatment (NAC = neoadjuvant chemotherapy.

	Before NAC Mean ± SD (range)	After NAC Mean ± SD (range)	Folder change	*P* value	Differences Mean ± SD (range)
Imaging evaluation (n = 48)					
3D-CEUS score	7.5 ± 1.6 (3–10)	3.3 ± 2.1 (0–8)	0.4	<0.0001	4.2 ± 2.3 (2–9)
DCE-MRI score	7.5 ± 1.2 (4–9)	3.3 ± 2.2 (0–8)	0.4	<0.0001	3.5 ± 1.6 (1–7)
Pathology evaluation (n = 40)					
MVD count (unit, 10^3^)	12 ± 3.3 (5.9–20)	7.8 ± 2.2 (3.4–13)	0.6	<0.0001	4.3 ± 2.4 (1.2–12)
VEGF score	5.6 ± 0.6 (4–6)	3.8 ± 0.9 (2–5)	0.7	<0.0001	1.8 ± 0.8 (1–4)

SD = standard deviation. DCE-MRI = dynamic contrast enhanced magnetic resonance imaging. 3D-CEUS = three dimensional contrast enhanced ultrasound. MVD = microvessel density. VGEF = vascular endothelial growth factor).

**Table 2:** Correlation between imaging score and microvessel quantification (n = 40, DCE-MRI = dynamic contrast enhanced magnetic resonance imaging.

	MVD	VEGF
Correlation before NAC		
3D-CEUS score	r = 0.729, *P* < 0.0001	r = 0.454, *P* = 0.0046
DCE-MRI score	r = −0.125, *P*= 0.4340	r = −0.247, *P* = 0.1235
Correlation after NAC		
3D-CEUS score	r = 0.549, *P* = 0.0006	r = 0.460, *P* = 0.0041
DCE-MRI score	r = −0.110, *P* = 0.4925	r = −0.054, *P* = 0.7365
Correlation of difference before and after NAC		
Δ3D-CEUS	r = 0.177, *P* = 0.2686	r = 0.408, *P* = 0.0108
ΔDCE-MRI	r = 0.085, *P* = 0.5954	r = 0.166, *P* = 0.2986

3D-CEUS = three-dimensional contrast enhanced ultrasound. MVD = microvessel density. VEGF = vascular endothelial growth factor. Δ3D-CEUS=score difference before and after treatment. ΔDCE-MRI = score difference before and after treatment).

**Table 3:** Correlation between imaging score and treatment response after NAC (n = 48, pCR = pathological complete response.

	pCR Mean ± SD (range)	non-pCR Mean ± SD (range)	*p* value	*r*	MHR Mean ± SD (range)	NMHR Mean ± SD (range)	*p* value	*r*
3D-CEUS score	0.5 ± 1.4 (0–4)	3.9 ± 1.7 (1–8)	0.0001	−0.573	2.1 ± 1.7 (0–4)	4.2 ± 1.9 (1–8)	0.0002	−0.541
DCE-MRI score	0.3 ± 0.7 (0–2)	3.9 ± 1.9 (0–8)	<0.0001	−0.615	1.5 ± 1.5 (0–4)	4.6 ± 1.6 (2–8)	<0.0001	−0.702
Δ3D-CEUS	8.1 ± 1.1 (6–9)	3.4 ± 1.5 (2–6)	<0.0001	0.649	6.5 ± 1.7 (4–9)	2.6 ± 0.7 (2–4)	<0.0001	0.863
ΔDCE-MRI	8.0 ± 1.1 (7–9)	3.5 ± 1.7 (1–7)	<0.0001	0.639	6.4 ± 1.6 (5–9)	2.7 ± 1.2 (1–5)	<0.0001	0.836

MHR = major histological response. NMHR = non-major histological response. DCE-MRI = dynamic contrast enhanced magnetic resonance imaging. 3D-CEUS = three dimensional contrast enhanced ultrasound. Δ3D-CEUS = score difference before and after treatment. ΔDCE-MRI = score difference before and after treatment).

**Table 4:** Performance of imaging change and microvessel change in predicting pCR and MHR (pCR = pathological complete response.

	Folder change	*P* value	AUC	Cutoff	Sensitivity	Specificity	PPV	NPV
pCR vs. non-pCR (n = 8 vs. n = 40)								
3D-CEUS score	0.1	0.0001	0.934 (0.824–0.968)	<1	88%	100%	100%	98%
DCE-MRI score	0.1	<0.0001	0.970 (0.875–0.998)	<1	88%	98%	88%	98%
Δ3D-CEUS	2.4	<0.0001	0.991 (0.909–1.000)	>6	88%	100%	100%	98%
ΔDCE-MRI	2.3	<0.0001	0.987 (0.903–1.000)	>5	100%	95%	80%	100%
MHR vs. NMHR (n = 20 vs. n = 28)								
3D-CEUS score	0.5	0.0003	0.810 (0.671–0.909)	<1	35%	100%	100%	68%
DCE-MRI score	0.3	<0.0001	0.906 (0.787–0.971)	<1	40%	100%	100%	70%
Δ3D-CEUS	2.5	<0.0001	0.993 (0.913–1.000)	>4	90%	100%	100%	94%
ΔDCE-MRI	2.4	<0.0001	0.982 (0.894–1.000)	>4	100%	93%	91%	100%
MHR vs. NMHR (n = 12 vs. n = 28)								
Δ MVD (unit, 10^3^)	1.3	0.2497	0.616 (0.449–0.765)	>4.32	58%	71%	47%	80%
Δ VEGF	1.3	0.0766	0.679 (0.512–0.817)	>3	18%	100%	100%	74%

MHR major histological response. NMHR = non-major histological response. AUC=area under the curve. PPV = positive predictive value. NPV = negative predictive value. DCE-MRI = dynamic contrast enhanced magnetic resonance imaging. 3D-CEUS = three dimensional contrast enhanced ultrasound. Δ3D-CEUS = score difference before and after treatment. ΔDCE-MRI = score difference before and after treatment. MVD = microvessel density. VGEF = vascular endothelial growth factor. ΔMVD = difference of MVD before and after treatment. ΔVEGF = difference of VEGF before and after treatment).

**Table 5:** 3D-CEUS and DCE-MRI scoring systems in benign and malignant breast tumors (3D-CEUS = three dimensional contrast enhanced ultrasound.

	0	1	2
3D-CEUS characteristics			
Peripheral vessels			
Presence and distribution	Absent	Present, not radial	Present, radial
Dilated degree and courses	Not coarse. not distorted	Not coarse, distorted	Coarse, distorted or not
Penetrating vessels	Absent	Running inside tumor	Running towards center
Rim perfusion	Absent	Thin/moderate	Coarse
Intratumoral vessels	Absent	Thin/smooth	Coarse
DCE-MRI index			
Enhancement pattern	Homogeneous	Heterogeneous	Rim enhancement
TIC curve	Type I (persistent)	Type II (plateau)	Type III (wash-out)
Initial signal increase	Mild	Moderate	Significant
Peripheral vessels	Not rich	Richer	Richer and coarser
Penetrating vessels	None	Thin/moderate	Coarse/Encircling

DCE-MRI = dynamic contrast enhanced magnetic resonance imaging).
